# Comparative compositional and functional venomic profiles among venom specimens from juvenile, subadult and adult Russell’s viper ( *Daboia siamensis* ): correlation with renal pathophysiology in experimental rabbits

**DOI:** 10.1590/1678-9199-JVATITD-2021-0111

**Published:** 2022-04-04

**Authors:** Narongsak Chaiyabutr, Lawan Chanhome, Taksa Vasaruchapong, Panithi Laoungbua, Orawan Khow, Anudep Rungsipipat, Onrapak Reamtong, Visith Sitprija

**Affiliations:** 1 Queen Saovabha Memorial Institute, The Thai Red Cross Society, Pathumwan, Bangkok, Thailand.; 2 Center of Excellence for Companion Animal Cancer, Department of Veterinary Pathology, Faculty of Veterinary Science, Chulalongkorn University, Bangkok, Thailand.; 3 Department of Molecular Tropical Medicine and Genetics, Faculty of Tropical Medicine, Mahidol University, Ratchathewi, Bangkok, Thailand.

**Keywords:** Daboia siamensis, Venomics, Snake age, Renal pathophysiology, Rabbit

## Abstract

**Background:**

Eastern Russell’s viper (*Daboia siamensis*) is one of the most medically significant snakes responsible for the development of acute renal failure. However, variation of the clinical picture and renal pathophysiology following bites by young and adult *D. siamensis* have not been elucidated.

**Methods:**

In this study, we analyzed the venomic profiles of *D. siamensis* at different maturation stages of juvenile, subadult and adult groups. The same pooled venom from each group was subjected to enzymatic, electrophoretic and proteomic analysis, including sublethal toxicity (0.1 mg/kg iv.) examined on bodily functions by comparing the venom compositional and functional profiles among venom specimens from juvenile, subadult and adult *D. siamensis* by correlating them with the renal pathophysiology in experimental rabbits.

**Results:**

The comparative studies revealed that juvenile venom possessed higher phospholipase A_2_, metalloproteinase and serine proteinase levels, while subadult and adult venoms contained more L-amino acid oxidase, phosphodiesterase, the Kunitz-type serine protease inhibitor, disintegrin families and endothelial growth factor. An *in vivo* study revealed that the adult and subadult venoms caused persistent hypotension and bradycardia, while thrombocytopenia was a more characteristic effect of juvenile venom. All venom age groups showed significant reductions in renal hemodynamics and electrolyte excretions. The juvenile venom caused a higher tubulonephrosis lesion score than adult and subadult venoms.

**Conclusions:**

The *D. siamensis* venom shows an ontogenetic shift in its compositions and activities. Renal function alterations after envenomation depend on either the synergistic actions of different venom components or the disproportionate expression between the concentrations of enzymatic and non-enzymatic proteins in each age venom group. The high proportion of enzymatic toxin proteins in the juvenile venom results in greater nephrotoxicity.

## Background

Snakebites are designated as a health problem and a neglected tropical disease in many parts of the world by the World Health Organization. Russell’s viper, one of the most commonly encountered snakes, is a medically important species responsible for a substantial number of deaths in many countries. Phylogenetic analysis has indicated that Russell’s viper constitutes two distinct species, i.e. *Daboia russelli* in South Asia and *D. siamensis* in Southeast Asia. The development of acute kidney injury (AKI) after envenomation with *D. siamensis* is an important clinical event and is associated with signiﬁcant mortality [1]. Although the most common complication amongst lethal cases of *D. siamensis* bites is acute renal failure (ARF), its pathogenesis is not well understood. Earlier studies in Myanmar reported that the clinical picture observed following bites by young and adult *D. siamensis* snakes varied [2], and that over 50**%** of *D*. *siamensis* bites were from young snakes [3]. Some studies have described the biochemical and biological properties of *D. siamensis* venom, showing higher lethal potency and powerful coagulant and defibrinogenating activities in young snakes compared to adults, but an age-dependent variation in the venom components associated with AKI development has not yet been confirmed [4]. Variation in the snake venom composition may be accompanied by distinct protein and non-protein components with different structures and specific biochemical activities that lead to variability in clinical presentation.

The severity of symptoms after envenomation would be expected to depend on the toxic components present in the venom, their relative proportions, and the inoculated volume. However, the rationale for the observed ontogenetic changes remains obscure, as little is known about the different pathophysiological changes in renal functions after envenomation with *D. siamensis* and the differences in the protein abundance of its venom. The mechanisms of venom action within the body to induce AKI during envenomation from *D. siamensis* have not been determined, although several studies in other snake species have reported ontogenetic differences among venoms of the same species, including variations in the biological and biochemical features [4, 5, 6] differences in venom compositions [7, 8, 9, 10, 11], toxicity [4, 12, 13, 14] and enzymatic activity [8, 14]. The abundance of different toxic and non-toxic proteins in snake venom are influenced by many factors, including variations in taxonomy, age, sex, geography, diet and seasons [15, 16, 17]. 

The effects of either toxic or/and non-toxic proteins in *D. siamensis* venom on kidney functions have not yet been comprehensively determined, although the difference in symptomatology after envenomation by *D. siamensis* snakes has been described [4]. Knowledge of the venom compositions derived from proteomic analysis would serve as a starting point to improve the understanding of the venom complexity and variability, and when coupled with kidney (the target organ) function studies. The findings will contribute towards elucidating the clinical pathophysiology of *D. siamensis* envenomation. In addition, interspecific variation in the snake venom composition may be accompanied by a different protein antigenicity that leads to suboptimal immunoreactivity and weak neutralization by clinically used antivenoms as reported in other snake species [18, 19]. Thus, detailed characterization of the variations in venom protein profile in juvenile, subadult, and adult *D. siamensis* might shed some light on the requirement for using pooled venoms as a representative venom for antivenom production. 

Based on this information, further investigations are required on whether the venomics from *D. siamensis* of varying ages show different actions in both the functional and compositional profiles. Therefore, this study aimed to compare the compositional and functional profiles among venom specimens from juvenile, subadult and adult *D. siamensis* by correlating them with the renal pathophysiology in experimental rabbits. 

## Methods

### Animals

Adult male white New Zealand rabbits, weighing 2-3 kg, were used as experimental animals for this *in vivo* study. Animals were obtained from the Animal House, Queen Saovabha Memorial Institute (QSMI) and were housed in stainless-steel cages, where they received water and a standard diet *ad libitum*, and were exposed to a 12:12 h light: dark cycle, and maintained at a laboratory temperature of 26 ± 1 ^o^C. The animals were quarantined for 14 d before experiments. *In vivo* experiments were performed by the permission of the Ethics Committee of the QSMI Animal Care and Use (approval number QSMI-ACUC-03-2016) under the guideline of the National Research Council of Thailand. 

### Experimental design

*Snake and venom sample collections*

Russell’s viper (*D. siamensis*) snakes collected from the eastern regions of Thailand were kept in captivity at the Snake Farm of the QSMI, Thailand, maintained individually in plastic cages, and provided water *ad libitum* in the same animal care room in the Snake Farm. Once a month, the snakes were fed small rodents in proportion to their weight (10-20% of the snake’s body weight; BW). All snakes were maintained under a normal environmental temperature (average 27°C) and relative humidity (75%). Wild-caught *D. siamensis*, both male and female, were divided by length and body girth size into three size groups. The straight-line length measurement was snout-to-vent distance whereas body girth was measured at mid-body.

Each snake was categorized into one of three developmental stage groups (juvenile, subadult or adult) based upon its body length and girth, as follows: (i) a total length of 22-27.5 cm and girth of 3.1-4.0 cm represented a juvenile snake; (ii) a total length of 53-74 cm and girth of 4.8-5.7 cm indicated a subadult snake; and (iii) a total length of 76-127 cm and girth of 10-15.7 cm represented an adult snake. Each snake was weighed and measured for its body length and girth at the time of venom extraction. The venom pool from juvenile *D. siamensis* was obtained from 34 specimens collected after their first shedding and first feeding, at about 6 weeks old, while 12 and 85 snakes were used for the pooled venom from sub-adult and adult snakes, respectively. The pooled venoms from each venom group were lyophilized and stored at -20°C until use. The same pooled venom from each group was divided into two portions that were then used for the proteomic analysis and the *in vivo* physiological studies in rabbits.

### Venom and chemical analyses

*Isolation and characterization of venom compositions*

Venom compositions from adult, subadult and juvenile *D. siamensis* were analyzed in two ways. Firstly, the isolation and initial characterization of the venom composition and enzymatic activities were performed for phospholipase A_2_ (PLA_2_) and metalloproteinase (MP) as the dominant protein families and L-amino acid oxidase (LAAO) and phosphodiesterase (PDE) as minor protein families for comparative purposes. Secondly, venom proteomes were characterized and quantified by protein bands from Coomassie Brilliant Blue-stained Tris-Tricine sodium dodecylsulphate polyacrylamide gel electrophoresis (SDS-PAGE), and protein spots from two-dimensional gel electrophoresis (2D-GE). This second part of the venom analyses was performed on individual venom samples for protein extraction and quantification by proteomic analysis using mass spectrometry (MS) analysis. 

*Determination of venom enzymatic activities*

The enzymatic activities of crude venoms from the adult, subadult and juvenile *D. siamensis* were measured as previously described [20]. Briefly, PLA_2_ activity was evaluated using 3 mM 4-nitro-3-(octanoyloxy) benzoic acid as the substrate, where 1 unit (U) of PLA_2_ activity was defined as the amount of enzyme that caused a change in the substrate absorbance at 425 nm of 0.1 arbitrary units (AU), equivalent to 25.8 nmoles of chromophore release [21]. The proteolytic activity and inhibitor assay for metalloproteinase (MP) activity were determined using 2% (w/v) casein in 0.5 M Tris-HCl, pH 8.0, as the substrate. After stopping the reaction by the addition of 5% (w/v) trichloroacetic acid, the hydrolyzed peptides in the supernatant were quantified by the Folin Ciocalteau method [22]. One U of proteolytic activity was defined as the amount of enzyme hydrolyzing casein at an initial tyrosine formation rate of 1.0 µM/min. The indication for inhibition of venom proteolytic activity of metalloproteinase fraction was observed by pre-incubating the venom samples with 10 mM EDTA for 10 min. The LAAO activity was ascertained as reported in Worthington Enzyme Manual [23], where 1 U of LAAO activity was defined as the amount of venom that caused an increase of 0.001 AU at 426 nm per minute. The PDE activity was measured using bis (p-nitrophenyl) phosphate as the substrate as reported [24], where 1 U of PDE activity was defined as the amount of enzyme that caused an increase of 0.001 AU at 440 nm per minute.

*Venom protein analysis by one-dimensional SDS-PAGE*

Each 30 µg (w/v) of venom sample was separated by 12% (w/v) SDS-PAGE according to a modified method of Laemmli [25]. Electrophoresis (resolving gels 9 cm wide x 10 cm long x 0.1 cm deep) was performed at room temperature using 30 mA for 90 min in 25 mM Tris-glycine, pH 8.8. The protein bands were visualized by Coomassie G-250 solution (Bio-Rad, USA). For characterization of individual pooled venom by MS analysis, whole gel lanes of snake venoms were cut into small pieces and kept at -80 °C until used.

*Venom protein analysis by 2D-GE*

The venom proteins from juvenile, subadult, and adult *D. siamensis* were separated by 2D-GE as previously described by Berkelman and Stenstedt [26]. The first-dimensional isoelectric focusing (IEF) was performed using 150 µg of venom samples diluted in 125 µL of 60 mM DTT, 4% (w/v) CHAPS, and 0.5% (v/v) immobilized pH gradient (IPG) buffer loaded into the 7 cm IPG gel strip containing a linear IPG from 3 to 10 (Amersham Bioscience Inc). Electrofocusing was performed at 30 kVh using an IPG at 20 ^o^C according to the manufacturer’s instructions.After IEF, the IPG gel strip was transferred to the second dimensional SDS-PAGE (12% polyacrylamide resolving gel) and subjected to electrophoresis as described in section 5.3.3. Protein spots were visualized by Coomassie Blue R-250 staining. 

*Venom protein analysis by mass spectrometry*

The MS protein profiles of each pooled venom from adult, subadult and juvenile *D. siamensis* were obtained as follows. The proteins (30 µg) were separated by SDS-PAGE (section 5.3.3), and the entire gel lane was excised and subdivided into bands. Each band was sequentially destained by soaking in 50% (v/v) acetonitrile (ACN; Merck, Germany) in 50 mM ammonium bicarbonate until colorless, reduced by 4 mM dithiothreitol (Merck, Germany), alkylated by 250 mM iodoacetamide (Merck, Germany), dehydrated in 100% (v/v) ACN, and dried at room temperature. Tryptic digestion was performed by adding trypsin solution (Sigma-Aldrich, USA, T6567) at a 1:100 (v/v) ratio, followed by overnight incubation at 37 °C. The digested peptides were extracted in ACN for 15 min, after which the supernatant was collected and dried using a centrifugal concentrator (TOMY, Japan). The peptides were resuspended in 0.1% (w/v) formic acid (Sigma-Aldrich, USA) and subjected to an Ultimate® 3000 Nano-LC system (Thermo Scientific, USA) controlled by the software Chromeleon™, Version 7.2 (Thermo Scientific, USA). A microTOF-Q II (Bruker, Germany) was coupled online with the LC systems. Sample acquisition was controlled by HyStar™ Version 3.2 (Bruker, Germany). 

The data were processed and converted to mascot generics files (mgf) using the software Compass Data Analysis™, Version 3.4 (Bruker, Germany) and screened against the NCBI Chordata database using Mascot Daemon software (Matrix Science, USA). Only proteins at a 95% significance threshold are reported in this paper. The exponentially modified protein abundance index (emPAI) was used for semi-quantification [27]. Proteins with a more than two-fold difference in at least two biological replications were reported as differential proteins. 

### *In vivo* studies of nephrotoxicity

*Animal preparation*

Experiments were performed on adult male white New Zealand rabbits. The day before the experimental study, the animal was deprived of food but not of water for 12 h prior to the study. After being anesthetized with sodium pentobarbital (50 mg/kg) by intravenous (IV) injection, the animal was tracheotomized to free the airway with an endotracheal tube. The jugular vein was cannulated with polyethylene tubes (PE 90) for infusion of the solution for renal clearance studies. The carotid artery was cannulated with a PE 90 tube for collection of blood samples and recording of the blood pressure and heart rate (HR) (Polygraph Model 79, Grass Instruments Co.) The left ureter was cannulated with a polyvinyl catheter (i.d 1.19 mm and o.d 1.8 mm) via a retroperitoneal approach for urine collection.

*Venom dose optimization*

Previous studies revealed that the lyophilized *D. siamensis* venom dose that caused the death of 50% of either experimental dogs or rabbits (LD_50_) was 0.5 mg/kg BW after intravenous injection (IV) [28 29]. However, in preliminary experiments using a single venom dose of either 0.1 or 0.5 mg/kg BW, rabbits injected with 0.1 mg/kg BW showed systemic and kidney function alterations, whereas those receiving 0.5 mg/kg BW generally died within a few minutes or hours after venom administration. This short survival time precluded adequate assessment of the changes in kidney functions, and thus the venom was administered at 0.1 mg/kg BW in this study as a compromise to provide the best combination of renal damage (assessed histologically) and a survival time of at least 3-4 h. Moreover, the IV administration of venom at 0.1 mg/kg BW produced minimal hemodynamic alterations whereas at higher doses the hypotension was progressive, leading to cardiovascular collapse and, in some instances, rapid death. 

*Determination of renal functions*

On the day of experiments, an anesthetized rabbit in each group was prepared for determining the effect of venom injection on renal functions. An injection of priming dose solution (0.5 mL/kg body weight) containing 5% inulin (In) and 1.2% *p*-amino hippuric acid (PAH) in 0.15 M NaCl, pH 7.4, was administered through the jugular vein catheter and then followed by the continuous infusion of the sustaining solution containing 0.5% inulin and 0.12% PAH in 0.15 M NaCl at a rate of 0.5 mL/min using a peristaltic pump (EYELA Microtube pump MP-3 Tokyo Rikakikai Co.Ltd.) throughout the experimental periods. After 30 minutes of equilibration time, the control period for kidney clearance studies and general circulation measurements were begun before pretreatment with *D. siamensis venom*. After completion of control measurements, three groups of male white New Zealand rabbits (four rabbits/group) were injected with specified lyophilized venom (0.1 mg/kg BW, IV) in 1 mL of 0.15 M NaCl, as the venom from juvenile, subadult, and adult *D. siamensis*, respectively, whilst the fourth control group received 1 mL of 0.15 M NaCl without any venom. In each group, the renal functions were assessed by the renal clearances of both In and PAH. All changes in renal clearance and general circulation were recorded at 5, 10, 30, 60, 90 and 120 min after envenomation. The urine sample was collected after envenomation along with arterial blood collection at the midpoint of the urine collection in each period. The renal hemodynamics, including mean arterial blood pressure (MAP), HR, packed cell volume (PCV), urine flow (UF), Inulin clearance, PAH-clearance, and urine electrolytes excretion, were determined. Evaluation of haematological parameters was performed in each period of study after envenomation. After the 3-hour period of the experiment, animals were euthanized with a high dose of pentobarbital sodium, after which both kidneys were removed and immediately immersed in the appropriate fixative for further tissue processing in histological analysis.

*Chemical analysis*

The In concentration in both the plasma and urine was determined by the modified anthrone method [30]. The determination of PAH concentration in the plasma and urine was performed by the Bratton and Marshall method as reported [31]. The Na^+^ and K^+^ ion concentrations were determined by flame photometry (Flame Photometers, Laboratory Instrument, BWB Technologies UK Ltd.), while the osmolality was measured using an osmometer (Fiske^®^ Micro-osmometer Model 210, Fiske^®^ Associates, Norwood, Massachusetts, 02062, USA). The Cl^-^ ion concentration was determined using a chloridometer (Chloride Analyzer 925, Corning Ltd.), plasma urea was measured by a spectrophotometer [32], plasma creatinine by the alkaline picrate method [31] and the concentration of plasma symmetric dimethylarginine (SDMA) was determined by the SDMA Test (Catalyst SDMA Test, IDEXX Laboratories, Inc. USA). 

*Calculation of renal functions*

The study of both In and PAH clearances was performed as previously described [28]. Renal clearance (C) was calculated from C = UV/P (U is the urine concentration, V is the UF rate, and P is the plasma concentration) using the plasma and urine In and PAH levels for each period. Inulin clearance (Cin) was used to estimate the glomerular filtration rate (GFR), while the PAH clearance (C_PAH_) was employed to estimate the effective renal plasma flow (RPF). Effective renal blood flow (RBF) was calculated as follows: RBF =ERPF x 100/100 - PCV; Filtration fraction (FF)=GFR x100/ERPF; Osmolar clearance (Cosm) = Uosm xV/Posm. The fractional sodium (FENa^+^), potassium (FEK^+^), and chloride (FECl^-^) excretions were calculated as CNa^+^/Cin, CK^+^/Cin, and CCl^-^ /Cin, respectively.

### Determination of hematological parameters

After envenomation in each study period of each group, blood samples were collected into tubes with K_3_EDTA anticoagulant for determination of the hematological parameters: red blood cells (RBC), hemoglobin (Hb), mean corpuscular volume (MCV), leukocytes, platelets, neutrophils, lymphocytes, and monocytes using an Auto-Hematology Analyzer (Mindray BC-5000 Vet, Mindray Biomedical Electronics Co. Ltd. Nanshan Shenzhen, China).

### Histopathological studies

Following the injection of rabbits as detailed in the section of venom dose optimization, both kidneys from rabbits in each group were dissected and cut sagittally before fixation in 10% (v/v) neutral buffered (pH 7.2) formalin solution for 48 h, dehydrated in a graded ethanol series, treated in an automated tissue processer, and embedded in paraffin wax. Then, 4-μm-thick kidney paraffin sections were cut, mounted and stained with Hematoxylin and Eosin (H&E) and Periodic Acid-Schiff, (PAS). The tissue sections were evaluated under light microscopy by a board-certified veterinary pathologist blinded to the treatment. Histopathological lesions of the glomeruli and proximal and distal tubules of the renal cortex and renal collecting ducts of the medullar part were examined covering at least 10 HPF areas in each section and semiquantitatively scored as not remarkable (0), mild (1), moderate (2) or severe (3) degree.

### Statistical analysis

The effects of *D. siamensis* venom on renal function alterations are expressed as the mean ± one standard deviation (SD). One-way ANOVA with repeated measures was used with Bonferroni’s *post-hoc* test to compare the number of changes among time points after the venom treatment within the same group. Significance was defined as p < 0.05. All data were analyzed by GraphPad Prism 5 for Windows (GraphPad Software, San Diego, CA, USA*).*

## Results

### Characterization and enzyme activity of *D. siamensis* venoms

**Figure 1.**
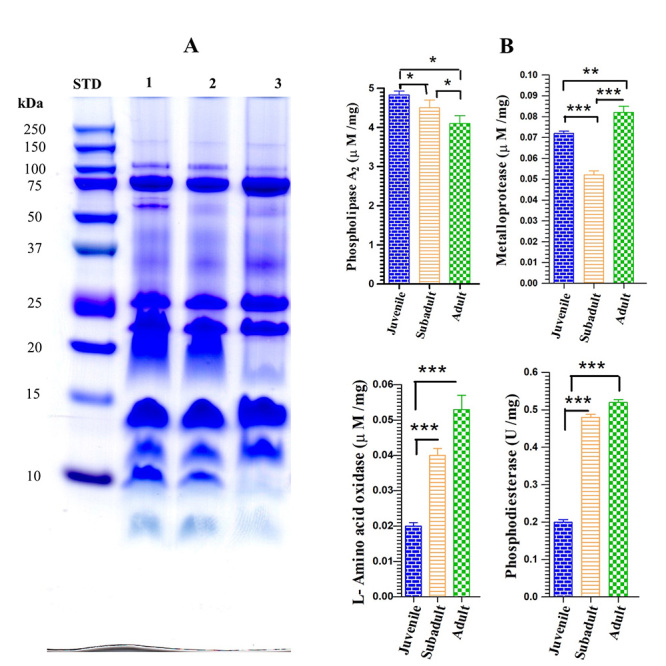
Characterization and enzyme activity of adult, subadult and juvenile *D. siamensis* venoms. **(A)** Representative SDS-PAGE patterns of protein bands (30 µg total protein/lane) from adult (lane 1), subadult (lane 2) and juvenile (lane 3) *D. siamensis* venom. **(B)** Comparative mean values of enzyme activities of PLA_2_ (µM/mg protein), metalloprotease (µM/mg protein), LAAO, (µM/mg protein) and PDE (U/mg protein) in the venom from adult, subadult, and juvenile *D. siamensis*. Values are presented as the meansSD for 3 measurements/venom group. Data for comparing each component enzyme activity among venom groups were analyzed using unpaired *t*-test indicated *p < 0.05, **p < 0.01, ***p < 0.001.

The molecular mass of venom proteins was accessed qualitatively using 12% SDS-PAGE under non-reducing conditions. The total number of protein bands varied between the adult, subadult and juvenile venoms (Figure 1A). The pattern of protein bands from the adult *D. siamensis* venom was broadly similar to that of the subadult venom (the stronger adult venom band at ca. 65 kDa), but juvenile *D. siamensis* venom had a larger proportion of high-molecular-weight protein bands (> 10 kDa) than those of adult and subadult. 

The PLA_2_ enzymatic activity was significantly higher in juvenile venom (p < 0.05) than those of subadult and adult venom, while the LAAO and PDE enzyme activities of juvenile venom were markedly lower (p < 0.001) in comparison to subadult and adult venoms. The snake venom metalloprotease (SVMP) activity in adult venom was significantly higher than those of juvenile and subadult venoms (Figure 1B).

### Comparative 2D-GE analysis of the protein family compositions of *D. siamensis* venoms

**Figure 2.**
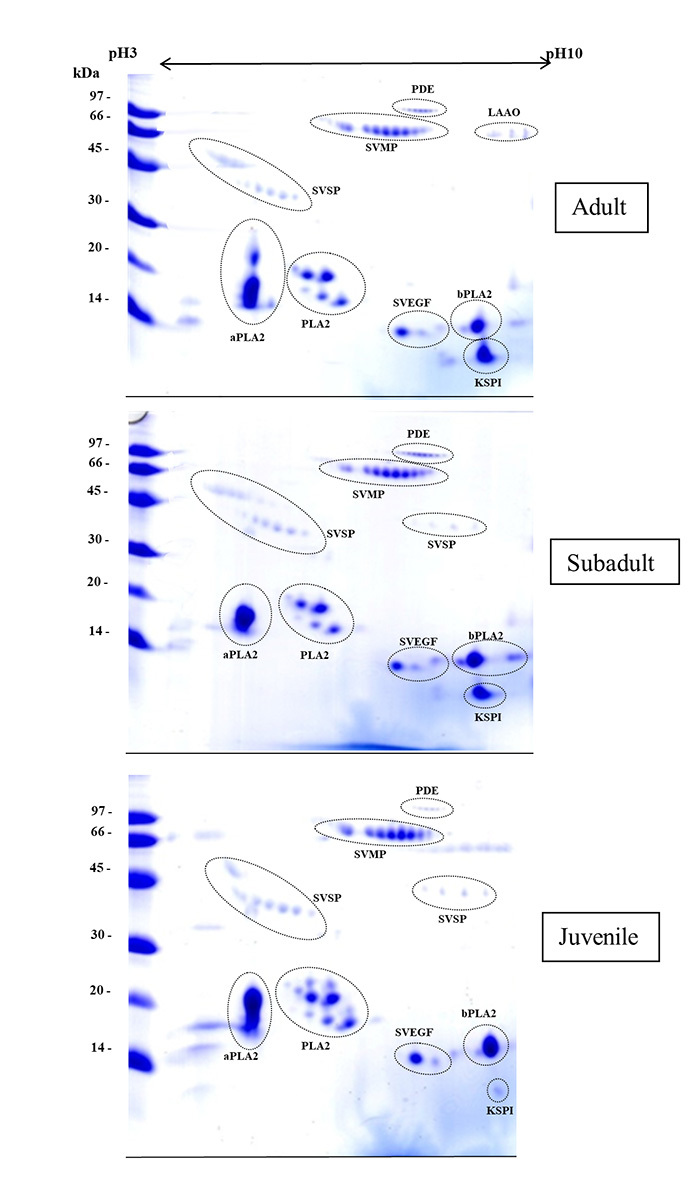
Comparative 2D-GE gel analysis of the protein family compositions among adult, subadult, and juvenile *D. siamensis* venoms. Total proteins (150 µg) from each pooled venoms group were isoelectrically focused (pI range 3-10) followed by SDS-PAGE resolution and Coomassie blue staining. Spots corresponding to protein molecules are encircled. Molecular mass markers (in kDa) are indicated at the left of each gel.

An ontogenetic variation in the composition of the pooled venoms is notable in 2D-GE profiles of *D. siamensis* at different ages (Figure 2). The 2D-GE images demonstrated differences in the number and intensity of protein spots in the juvenile, subadult and adult venoms, based upon the protein location in the gel (molecular mass and pI). These results were similar to those reported for *D. siamensis* specimens from Myanmar [33] and Taiwan [34]. Thus, the identification of protein spots in the venom specimens of this study was based on those studies. The separated protein spots from *D. siamensis* venoms in this present study were arranged into eight groups, which are typically abundant in *D. siamensis* venoms. The following protein families were identified: snake venom serine protease (SVSP), SVMP, basic and acidic PLA_2_, LAAO, PDE, snake vascular endothelial growth factors (SVEGFs) and Kunitz-type serine protease inhibitor (KSPI). However, the presence of low-molecular-mass (10 kDa) proteins, such as disintegrin, were not evaluated since this 2D-GE analysis only resolved venom proteins of more than 10 kDa. 

The gel images of three venom groups mostly had the same protein composition reported in previous studies [33, 34], which serves as a control that the venoms are not radically different from previously investigated venoms. The KSPI spots were abundant in adult venom but less abundant in sub-adult and undetectable in juvenile venoms. The volume of SVEGF spots from the juvenile venom was lower than in the subadult and adult *D. siamensis* venoms. Many venom PLA_2_ spots were located in the acidic PLA_2_ region in all three groups. The venom protein spots for LAAO were apparent in the adult venom and few in juvenile venom but not in subadult venom, suggesting that the concentration of LAAO in venom increased during ontogenetic development. 

### Proteomic profiles

*Venn diagram analysis*

**Figure 3.**
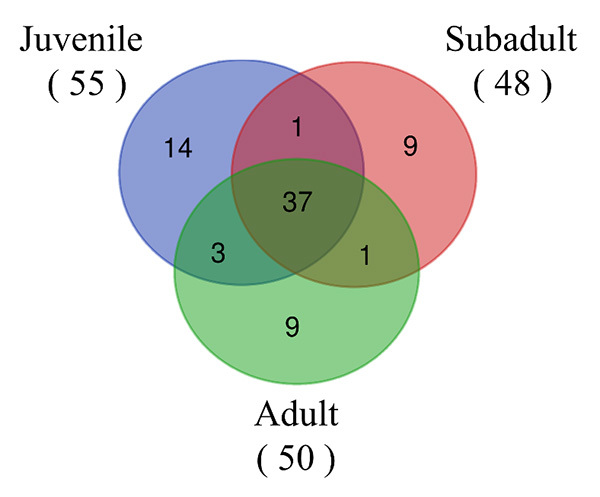
Venn diagrams analysis. This figure shows overlapping proteins in relation to exclusivity and interconnection of proteins found among juvenile, subadult, and adult *D. siamensis* venom.

Proteome alignment profiles are represented by Venn diagrams to depict the co-expressed and uniquely expressed proteins in the adult, subadult and juvenile *D. siamensis* venoms (Figure 3). A total of 74 proteins were identified in the venom specimens from the three age groups of snakes, of which 37 proteins (50%) were commonly found in the venom from all three age groups. Considering only the proteins differentially or uniquely expressed in the groups of snakes (quantified and/or only identified), the number of identified proteins overlapping between juvenile and subadult venoms was 38 (51.3%), of which one was not found in adult venom and 14, 9 and 9 proteins were observed in the juvenile, subadult, and adult venoms only, respectively. The number of identified proteins overlapping between juvenile and adult venom was 40 (54.1%), of which 3 were found only in juvenile and adult venom. The number of identified proteins overlapping between subadult and adult venom was 38 (51.4%), of which one was not found in juvenile venom. 

*Mass spectrometry analysis*

**Figure 4.**
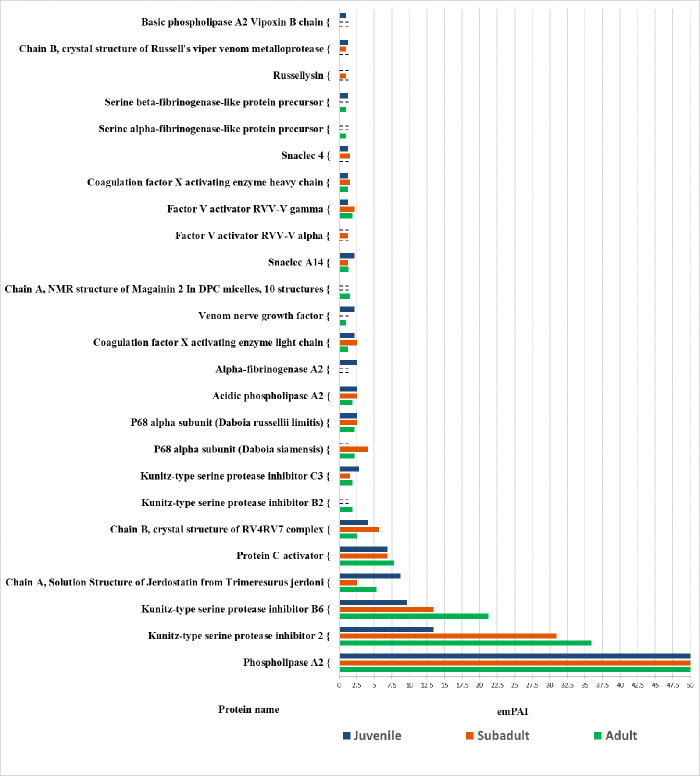
The abundance of the top-twenty most abundant venom proteins (as emPAI values) from juvenile, subadult and adult *D. siamensis*. - not detected.

Quantification of the *D*. *siamensis* venom proteins was performed using the emPAI data provided by the Mascot server. The emPAI values in this report were the mean of at least two biological replications, and proteins with a more than two-fold difference were reported as differential proteins. The top 20 most abundant proteins in the venom from juvenile, subadult and adult snakes are shown in Figure 4 and Table 1. According to this protein quantification, only venom proteins above the 95% significance threshold were reported by emPAI for label-free quantification. The emPAI values for PLA_2_ were extremely high in all three snake age groups, and represent the most abundant enzymatic proteins in the venom of *D. siamensis*. However, the emPAI values for PLA_2_ in juvenile venom (= 816) were nearly three-fold higher than in adult venom (= 266) (Table 1).

**Table 1.** The most common venom proteins (ranked by the emPAI value) from juvenile, subadult and adult *D. siamensis.*

Accession number	Protein	emPAI values		
		Juvenile	Subadult	Adult
P86529.1	PLA_2_ [*Daboia russelii*]	816.07	816.00	266.23
P00990.1	Venom basic protease inhibitor 2 [*Daboia siamensis*]	13.45	31.19	36.25
A8Y7P6.1	Trypsin inhibitor 6 [*Daboia siamensis*]	9.83	13.16	21.50
P33588.1	SVSP [*Agkistrodon bilineatus*]	6.90	6.90	7.91
A8Y7N6.1	Trypsin inhibitor 3 [*Daboia siamensis*]	2.84	1.57	1.79
P31100.1	Viperotoxin non-tox [*Daboia siamensis*]	2.61	2.49	1.97
Q9PRW3.1	Alpha-fibrinogenase A2 [*Crotalus atrox*]	2.57	N/A	N/A
AAB22478.1	Disintegrin (platelet aggregation inhibitor)-like MP [*Daboia russelii*]	2.13	2.57	1.33
P30894.1	Venom nerve growth factor [*Daboia russelii*]	2.13	0.58	1.12
P18965.2	Russell’s viper venom factor V activator gamma [*Daboia siamensis*]	1.31	2.10	1.87
AAB22477.1	MP with disintegrin (platelet aggregation inhibitor)-like and C-type lectin-like domains [*Daboia russelii*]	1.22	1.71	1.24
2E3X_B	Chain B, crystal structure of Russell’s viper SVMP [*Daboia siamensis*]	1.17	0.84	0.47
P14420.1	Basic PLA_2_ vipoxin B chain [*Vipera ammodytes meridionalis*]	1.04	N/A	N/A
A8Y7N4.1	Trypsin inhibitor 1 [*Daboia siamensis*]	0.90	N/A	0.92
A8Y7P1.1	Trypsin inhibitor B1 [*Daboia siamensis*]	0.41	0.37	0.37
A8Y7P2.1	KSPI B2 [*Daboia siamensis*]	0.41	N/A	1.82
Q9PT40.1	SVSP-like protein 2 [*Macrovipera lebetina*]	0.39	0.12	0.13
Q2ES48.1	KSPI 3 [*Daboia russelii*]	0.37	N/A	N/A
P04084.3	Vipoxin acidic component [*Vipera ammodytes meridionalis*]	0.27	0.59	0.56
E0Y419.1	Beta-fibrinogenase [*Macrovipera lebetina*]	0.27	0.26	0.28
P0DKR3.1	Acidic PLA_2_ CbI alpha [*Pseudocerastes fieldi*]	0.25	0.26	0.28
E0Y420.1	SVSP [*Macrovipera lebetina*]	0.25	0.12	0.13
Q1RP79.1	Basic PLA_2_ chain HDP-1P [*Vipera berus nikolskii*]	0.24	0.23	N/A
P67861.2	SVEGF [*Daboia russelii*]	0.23	0.48	0.46
Q7T046.1	SV MP [*Macrovipera lebetina*]	0.15	0.15	0.17
P18964.1	Russell’s viper venom factor V activator alpha [*Daboia siamensis*]	0.13	1.31	1.05
Q8UVX1.1	SVSP gussurobin [*Gloydius ussuriensis*]	0.12	N/A	N/A
E5AJX2.1	SVSP nikobin [*Vipera berus nikolskii*]	0.12	N/A	0.13
ADW54332.1	Group III SVMP [*Echis ocellatus*]	0.10	N/A	N/A
Q4VM08.1	SVMP [*Macrovipera lebetina*]	0.05	0.05	0.05
Q7LZ61.2	Coagulation factor X-activating enzyme heavy chain [*Daboia siamensis*]	N/A	0.91	N/A
1WQ9_A	Chain A, Vascular endothelial growth factor [*Daboia russelii russelii*]	N/A	0.31	N/A
E0Y418.1	SVSP [*Macrovipera lebetina*]	N/A	0.12	N/A
CAJ01689.1	Group III SVMP [*Echis ocellatus*]	N/A	0.05	N/A
XP_007460206.1	Cytosolic PLA_2_ zeta [*Lipotes vexillifer*]	N/A	0.03	0.03

N/A: not applicable

The distinctive PLA_2_ components in both subadult and adult venoms were acidic PLA_2_, while the amount of PLA_2_ components in the basic and acidic PLA_2_ in the juvenile venom were nearly three-fold higher than in the subadult and adult venoms (Figure 4, Table 1). The serine protease inhibitor was also high in the venoms of all age groups, but the emPAI values for venom basic protease inhibitor 2 in both the adult (= 36) and subadult (= 31) venoms were nearly three-fold higher than in the juvenile venom (= 13) (Table 1). Moreover, the venom components for fibrinogenase and MP were also found in a high proportion in juvenile snake venom.

In the present study, the KSPIs were the most abundant non-enzymatic proteins in *D. siamensis* venoms (Figure 4), including basic protease inhibitors, trypsin inhibitors, and SVEGFs (Table 1). The emPAI values revealed that both adult and subadult venoms contained an abundance of KSPIs, basic protease inhibitors, and trypsin inhibitors, which were about two-fold higher than in juvenile venom. The relative abundance of factor X activating enzyme in juveniles was higher than those in the subadult and adult venoms, while a similar amount of factor V activators was apparent in the venom of all three snake age groups. The SVEGFs in adult and subadult venoms (emPAI values of 0.46-0.48) were about two-fold higher than in the juvenile venom (emPAI = 0.23) (Table 1). The emPAI values for some venom protein families - for example, alpha-fibrinogenase A2 (= 2.75), basic PLA_2_ vipoxin B chain (= 1.04), KSPI-3(= 0.37), SVSP gussurobin (= 0.12) and SVMP group III (= 0.10) - were identified only in the juvenile venom (Figure 4, Table 1). Moreover, SVMP was the second most abundant enzymatic protein family in *D. siamensis* followed by SVSP, whereas KSPI and Snaclec were found to be the second most abundant non-enzymatic protein class and appeared to be more abundant in juvenile specimens compared to adult and subadult venoms (Figure 4, Table 1). The total proteins identified from each pooled venom of juvenile, subadult and adult are presented in the Additional file 1.

### *In vivo* study

*Effects of D. siamensis venom on general circulation, PCV, and plasma concentrations of creatinine, urea and SDMA*

**Figure 5.**
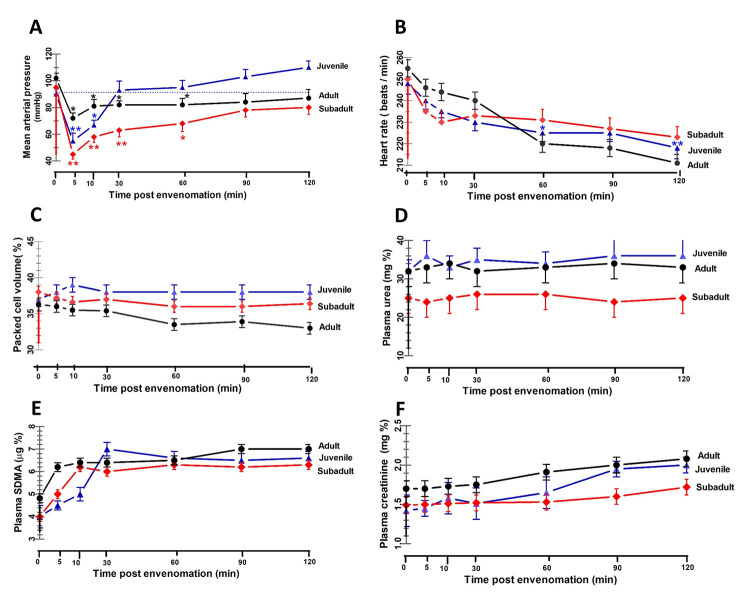
Progress of the **(A)** MAP, **(B)** HR, **(C)** PCV, and the plasma levels of **(D)** urea, **(E)** SDMA and **(F)** creatinine, in response to *D. siamensis* venoms in anesthetized rabbits. Three groups of four rabbits each were intravenously injected with *D. siamensis* venom (0.1 mg/kg BW) from juvenile (blue triangles), subadult (red diamonds) and adult snakes (dark circles). The points represent the mean ± SD (vertical bars). Significant difference at *p < 0.05 and **p < 0.01 level between the internal control and each post-envenomation time within each group (n = 4).

The *D. siamensis* venom (sublethal dose 0.1 mg/kg BW, IV) from all three age groups caused immediate depressor responses in the MAP and reached a maximal decrease within 5 minutes (P < 0.05; Figure 5A). This transitory decrease in the MAP was followed by gradual recovery (10 to 120 minutes) to basal levels. A different extent of compensation during the rise in MAP in the second phase after envenomation was evident between venoms from the three age groups. After administration of the juvenile venom, the initial sharp decrease in MAP was followed by a gradual recovery to pretreated levels during the ensuing 30 minutes and then increased above the control value at 60, 90 and 120 min after envenomation. In contrast to the effect of juvenile venom, the stepwise rise in the MAP in the second phase after envenomation with either subadult or adult venom tended to be less pronounced compared to that with juvenile venom, and the MAP remained significantly below the pretreatment values throughout the 120-minute experimental period. Changes in MAP occurred in a biphasic response in all venom groups, while the HR fell from the control value at each point throughout the 120-minute period after envenomation (Figure 5B). Despite the marked fall in MAP, there was no significant change in the HR at the point when the pressure had its maximal decrease, indicating that a direct cardiac effect was unlikely to be responsible for acute hypotension. There was no significant change in the PCV after envenomation in all three venom groups (Figure 5C). All *D. siamensis* venom groups induced a non-significant increase in the plasma creatinine and SDMA levels compared to the pretreated value, but the plasma urea levels were not different when compared to the pretreated value (Figures 5D-5F).

*Effects of D. siamensis venom on renal hemodynamics*

**Figure 6.**
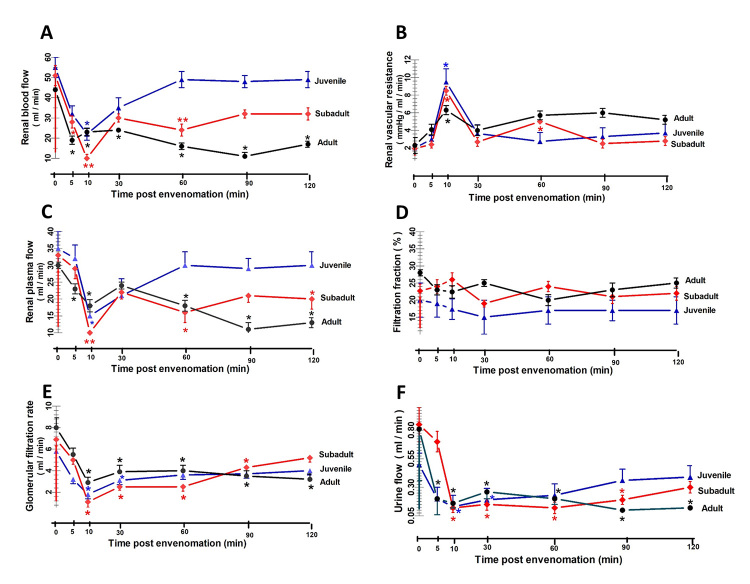
Effects of *D. siamensis* venoms on renal hemodynamics. Changes in the **(A)** RBF, **(B)** RVR, **(C)** RPF, **(D)** FF, **(E)** GFR and **(F)** UF in anesthetized rabbits in response to adult, subadult and juvenile *D. siamensis* venom. Three groups of four rabbits each were intravenously injected with *D. siamensis* venoms (0.1 mg/kg BW) from juvenile (blue triangles), subadult (red diamonds) and adult snakes (dark circles). The points represent the mean ± SD (vertical bars). Significant difference at *p < 0.05 and **p < 0.01 level between the internal control and each post-envenomation time within each group (n = 4).

The administration of *D. siamensis* venoms from all three age groups resulted in an immediate decrease in both the effective renal blood flow (RBF) and effective RPF (Figures 6A and 6C), which reached a maximal decrease within 10 minutes (p < 0.05), followed by gradual recovery to control levels, while the renal vascular resistance (RVR) increased approximately three-fold to a maximal level within 10 minutes (p < 0.05), followed by a gradual decline (10-120 min) to control levels after envenomation (Figure 6B). After the initial decreases in both the RPF and RBF in animals treated with either adult or subadult venom, they tended to increase in a stepwise fashion but remained significantly (p < 0.05) below (at 26% and 28%, respectively) the pretreated values at 60 minutes, in contrast to the results from the juvenile venom, which showed no significant changes compared to the control while the RBF returned towards pretreatment levels even though the MAP remained above the control level after envenomation. The administration of *D. siamensis* venoms significantly decreased (p < 0.05) the GFR and the urine flow rate (UF) throughout the experimental period in all three venom groups (Figures 6E and 6F). The filtration fraction (FF) showed no significant alteration after envenomation in all three age groups throughout the study (Figure 6D). 

*Effects of D. siamensis venom on the plasma electrolyte concentrations, fractional electrolytes excretions, and osmolar clearance*

**Figure 7.**
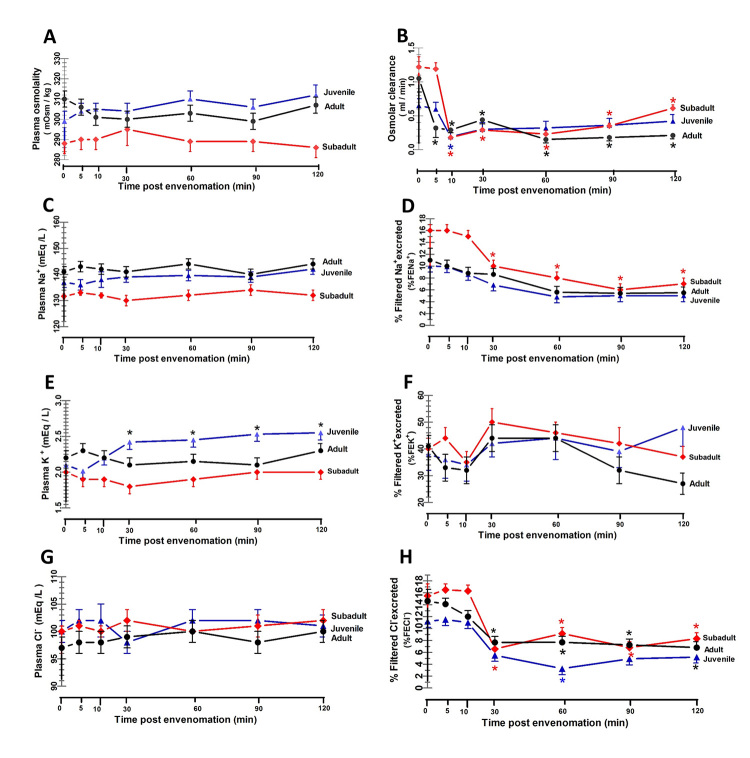
Changes in plasma concentrations and fractional excretions (FE) of Na^+^, K^+^ and Cl^-^, including the plasma osmolality and osmolar clearance, after *D. siamensis* venom injection into anesthetized rabbits. Three groups of four rabbits each were intravenously injected with *D. siamensis* venom (0.1 mg/kg BW) from juvenile (blue triangles), subadult (red diamonds) and adult snakes (dark circles). The points represent the mean ± SD (vertical bars). Significant difference at *p < 0.05 and **p < 0.01 between the internal control and each post-envenomation time in each age group (n = 4).

Administration of either subadult or adult *D. siamensis* venom caused no significant changes in the plasma concentrations of sodium ions (P_Na_^+^), chloride ions (P_Cl_^-^) or plasma osmolality (Posm) (Figures 7A, 7C and 7G) compared to the pretreated values throughout the study period, whereas the plasma concentration of potassium ions (P_K_^+^) was significantly increased (p < 0.05) at 30, 60, 90 and 120 minutes after juvenile venom administration (Figure 7E). The *D. siamensis* venom from all three age groups caused a significant decrease in GFR and UF. The reduced GFR after envenomation in all three age groups resulted in decreased filtered loads of Na^+^, K^+^ and Cl^-^ at levels equivalent to the decreased excretion of Na^+^, K^+^, and Cl^-^. Therefore, the urinary fractional excretion of Na^+^ (%FE_Na+_), Cl^-^ (%FE_Cl_-) (Figures 7D and 7H) and osmolar clearance (Figure 7B) started to decrease in the first 30 minutes. These effects were observed throughout the 120-minute study period in all three age groups, whereas the %FE_K+_ started to increase in the first 10 minutes and then tended to increase throughout the experimental period after envenomation in all three age groups (Figure 7F). 

*Comparative effects of D. siamensis venom on kidney histology*

**Figure 8.**
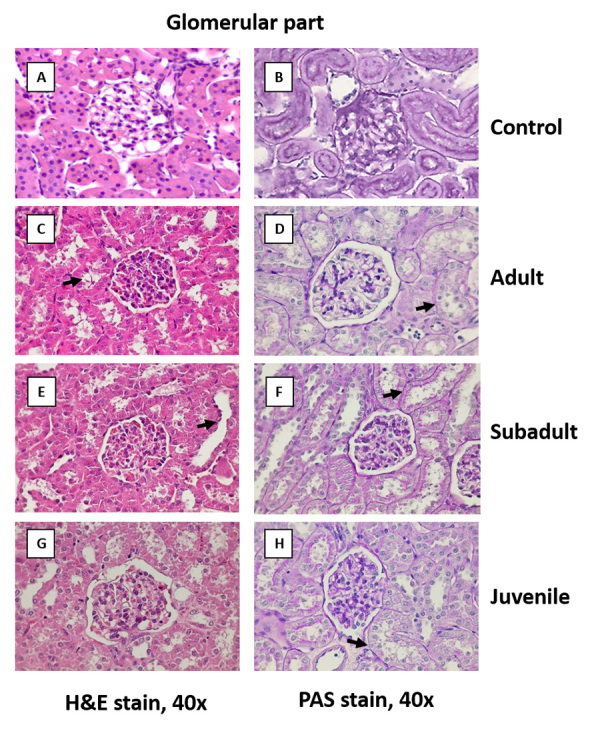
Comparative effects of *D. siamensis* on the kidney histology of the glomerular part. Representative photomicrographs (40x magnification) of the glomerular portion of kidney sections stained with **(A, C, E, G)** H&E and **(B, D, F, H)** PAS showing the **(A, B)** control kidney, and after envenoming with **(C, D)** adult, **(E, F)** subadult and **(G, H)** juvenile *D. siamensis* venom. Note: normal brush border of the proximal convoluted tubules of the control kidney and widening of the tubular lumen (black arrows) in venom-treated rabbit kidney. There was no remarkable lesion of the glomerular in either control or venom-treated rabbit kidneys.

**Figure 9.**
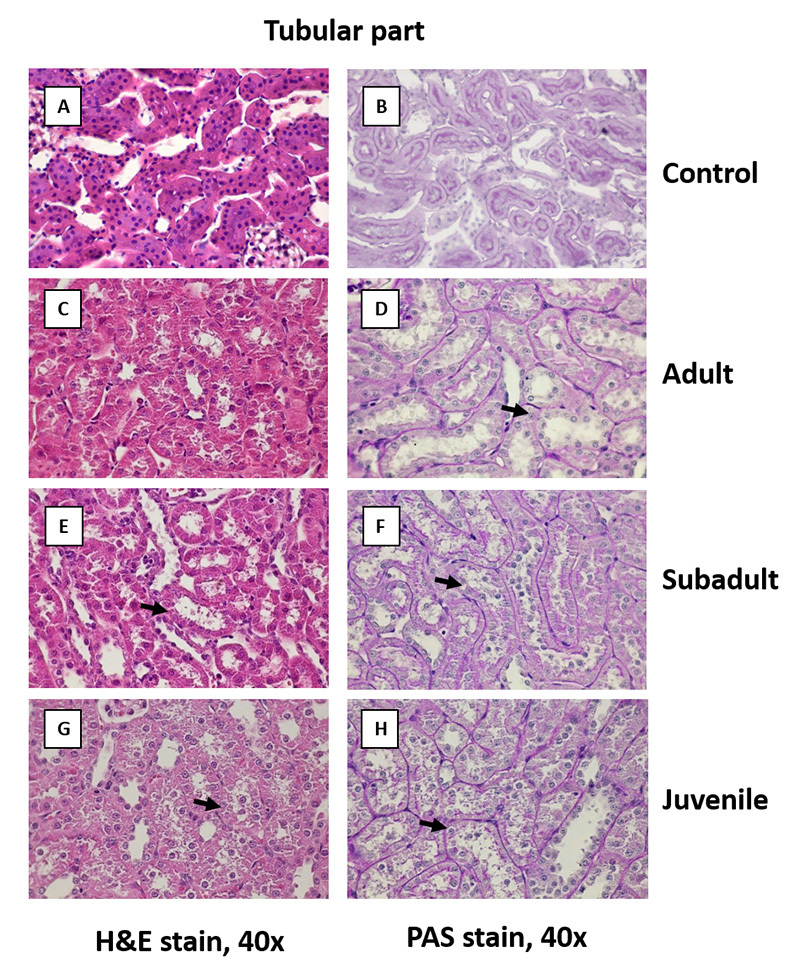
Comparative effects of *D. siamensis* on the kidney histology of the tubular part. Representative photomicrographs (40x magnification) of the tubular portions of rabbit kidney sections stained with **(A, C, E, G)** H&E and **(B, D, F, H)** PAS, of **(A, B)** control and after envenomation with **(C, D)** adult, **(E, F)** subadult and **(G, H)** juvenile *D. siamensis* venom. Note: control kidney shows a normal brush border in the proximal convoluted tubule. The adult and subadult *D. siamensis* venom induced mild diffuse acute tubulonephrosis in both the proximal and distal convoluted tubules (black arrows) (score 0.13 each), while juvenile venom induced mild diffuse acute tubulonephrosis of the proximal and distal convoluted tubules (score 0.47 and 0.27, respectively).

**Figure 10.**
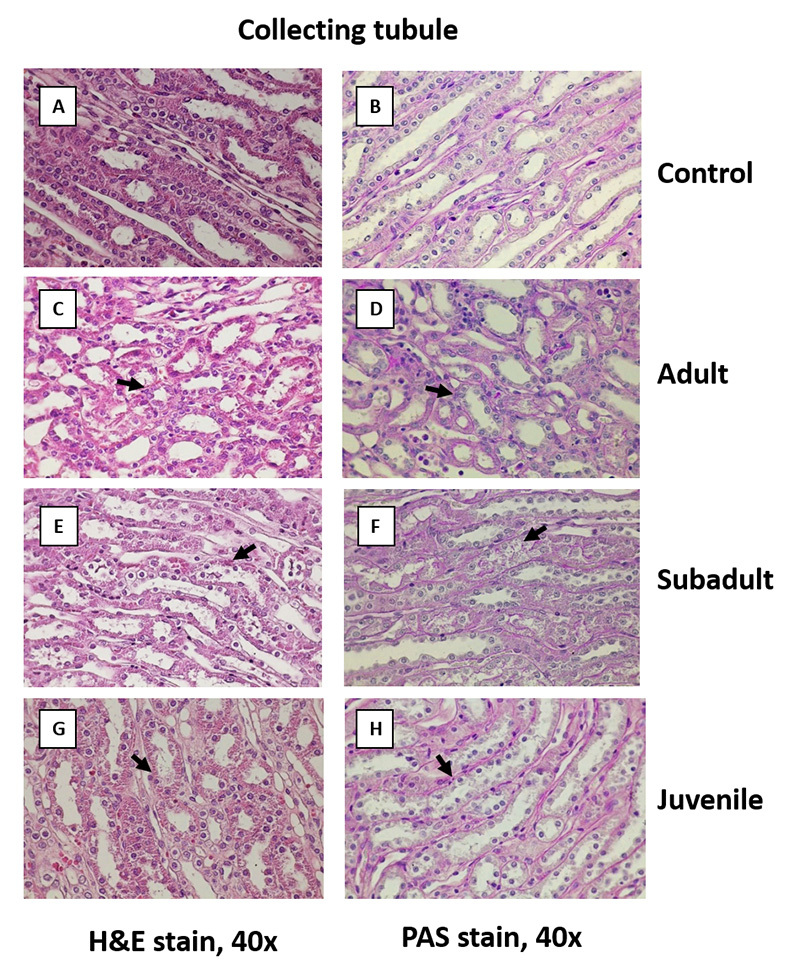
Comparative effects of *D. siamensis* on the kidney histology of collecting tubule. Representative photomicrographs (40x magnification) of the collecting duct of rabbit kidney sections stained with **(A, C, E, G)** H&E and **(B, D, F, H)** PAS of **(A, B)** control and after envenomation with **(C, D)** adult, **(E, F)** subadult and **(G, H)** juvenile *D. siamensis* venom. Note: control, as well as adult, and subadult envenomated kidneys showed no remarkable lesion of collecting tubules, whereas juvenile venom induced mild diffuse acute tubulonephrosis of the collecting tubules (black arrows) (score 0.33).

In the control group, animals showed a normal renal histology with no remarkable lesions in either the glomerular or tubular part (panels A and B in Figures 8 - 10). In the adult and subadult venom groups, the rabbit kidney showed moderate glomerular congestion (score 0.67 each; Figures 8 C - 8 F); the juvenile venom group also showed moderate congestion of the glomerular part (score 0.67; Figures 8G and 8H).

In the renal tubules, the adult venom induced mild diffuse acute tubulonephrosis mainly in the proximal and distal convoluted tubules (score 0.13 each). The affected tubules had a diffuse cloudy swelling of the cytoplasm with small homogeneous eosinophilic hyaline droplets. Some cells were detached from the tubular basement membrane and contained small round dense nuclei (Figures 9C and 9D). The subadult venom group showed mild diffuse acute tubulonephrosis, especially in the proximal or distal convoluted tubules (score 0.13 each; Figures 9E and 9F). The juvenile venom group showed mild diffuse acute tubulonephrosis of the proximal convoluted tubules (score 0.47) and diffuse acute tubulonephrosis of the distal convoluted tubules (score 0.27; Figures 9G and 9H).

With respect to the renal collecting tubules, the adult and subadult venom provoked no remarkable lesion of the collecting tubules (Figures 10C to 10F), while the juvenile venom induced mild diffuse acute tubulonephrosis of the collecting tubules (score 0.33; Figures 10G and 10H).

### Hematological studies

Comparison of the effects of venoms from adult, subadult, and juvenile *D. siamensis* on the hematological parameters (Table 2) revealed a decreased platelet count in all three age groups after envenomation. However, the platelets count was significantly lower in the juvenile venom group (p < 0.01) than in the subadult and adult venom groups. All three venom groups increased the MCV, but a particularly significant rise was provoked by the juvenile venom (p < 0.001) at 90 minutes after envenomation. White cell differential counts for monocytes tended to decrease after envenomation in all three venom groups.

**Table 2.** Effects of *D. siamensis* venom on the hematological parameters of experimental rabbits at 5, 10, 30, 60, 90 and 120 minutes after venom administration.

	Time post-experimental envenomation (min)					
Variable	Control	5	10	30	60	90	120
**RBC (10^6^/µL)**							
Juvenile	5.50 ± 0.50	5.34 ± 0.37	5.26 ± 0.49	5.20 ± 0.49	5.10 ± 0.37	5.32 ± 0.38	5.31 ± 0.61
Subadult	5.31 ± 0.10	5.04 ± 0.03	4.79 ± 0.02	4.84 ± 0.05	4.60 ± 0.03	4.63 ± 0.10	4.92 ± 0.18
Adult	5.10 ± 0.90	4.94 ± 0.65	4.85 ± 0.67	4.77 ± 0.85	4.51 ± 1.05	4.45 ± 1.09	4.47 ± 1.10
**Hb (g/dL)**							
Juvenile	11.50 ± 0.91	11.22 ± 0.55	11.20 ± 0.82	10.97 ± 0.81	10.83 ± 0.85	11.07 ± 0.72	11.00 ± 0.93
Subadult	11.85 ± 0.52	11.35 ± 0.29	10.85 ± 0.06	10.90 ± 0.00	10.60 ± 0.23	10.60 ± 0.00	11.00 ± 0.12
Adult	11.20 ± 1.23	10.77 ± 0.97	10.57 ± 1.11	10.33 ± 1.3	9.70 ± 1.79	9.57 ± 1.89	9.63 ± 1.88
**MCV (fL)**							
Juvenile	66.4 ± 0.70	70.8 ± 1.91	75.2 ± 3.31^**^	73.8 ± 5.19^**^	71.6 ± 3.54^*^	74.9 ± 4.68^***^	70.7 ± 1.03
Subadult	69.8 ± 1.50	72.3 ± 3.09	74.7 ± 4.68	73.8 ± 2.31	74.9 ± 4.68	71.9 ± 2.83	72.0 ± 2.42
Adult	69.3 ± 3.79	72.0 ± 5.33	74.7 ± 6.90	72.0 ± 4.96	72.4 ± 4.42	74.7 ± 6.10	70.0 ± 3.20
**Platelet (10^3^/ µL)**							
Juvenile	303 ± 61	255 ± 87	208 ± 62	139 ± 29^**^	141 ± 56^**^	184 ± 71	127 ± 34^**^
Subadult	435 ± 52	404 ± 92	372. ± 79	356 ± 51	316 ± 27	301 ± 12	315 ± 19
Adult	538 ± 146	473 ± 133	442 ± 185	430 ± 143	374 ± 185	349 ± 158	319 ± 115
**Leukocyte (10^3^/ µL)**							
Juvenile	1.98 ± 0.32	1.62 ± 0.51	1.51 ± 0.46	1.46 ± 0.44	1.19 ± 0.26	1.12 ± 0.17	1.22 ± 0.29
Subadult	1.86 ± 0.15	1.84 ± 0.12	1.83 ± 0.09	1.69 ± 0.38	1.12 ± 0.14	1.35 ± 0.13	1.83 ± 0.03
Adult	2.27 ± 0.14	1.70 ± 0.35	1.44 ± 0.28	1.59 ± 0.69	1.32 ± 0.34	1.43 ± 0.33	1.59 ± 0.63
**Lymphocyte (%)**							
Juvenile	58.6 ± 18.60	58.2 ± 15.23	58.9 ± 14.22	58.6 ± 4.08	62.3 ± 9.80	65.7 ± 12.11	65.3 ± 14.78
Subadult	59.7 ± 3.93	55.5 ± 3.32	51.3 ± 2.71	49.4 ± 7.62	60.0 ± 4.00	48.7 ± 1.91	32.0 ± 2.12
Adult	60.5 ± 16.57	66.2 ± 9.90	72.0 ± 4.59	67.6 ± 9.95	70.0 ± 3.54	70.4 ± 1.94	67.9 ± 10.23
**Monocyte (%)**							
Juvenile	6.9 ± 1.17	6.3 ± 2.12	4.6 ± 1.61	4.3 ± 1.58	3.1 ± 0.32	2.0 ± 0.40	2.6 ± 0.29
Subadult	7.5 ± 0.12	6.5 ± 0.03	5.6 ± 0.17	5.0 ± 0.75	4.4 ± 0.46	4.9 ± 0.98	3.8 ± 1.62
Adult	2.8 ± 2.09	2.7 ± 1.99	2.6 ± 1.91	3.1 ± 2.84	2.9 ± 2.22	2.0 ± 1.54	1.7 ± 1.34
**Neutrophil (%)**							
Juvenile	25.9 ± 20.67	28.1 ± 16.62	28.4 ± 15.98	30.7 ± 6.04	29.2 ± 9.24	27.4 ± 11.58	28.0 ± 12.81
Subadult	26.5 ± 4.56	32.6 ± 3.09	38.8 ± 1.62	40.8 ± 5.89	31.4 ± 0.17	41.1 ± 1.27	58.4 ± 3.98
Adult	28.5 ± 20.47	22.8 ± 14.25	17.9 ± 7.62	23.8 ± 10.82	20.8 ± 3.81	22.2 ± 3.30	24.3 ± 8.70
							

Data are presented as the mean ± SD of four different animals in each group. *p*-values analyzed by repeated measures ANOVA with Bonferroni *post-hoc* test: *p < 0.05, **p < 0.01, ***p < 0.001, mean values of specified time period with respect to the control period in the same group (n = 4).

## Discussion

The purpose of this study was to compare the venom compositional and functional profiles among venom specimens from juvenile, subadult and adult *D. siamensis* by correlating them with the renal pathophysiology in experimental rabbits. Given that renal physiopathological alterations induced by ontogenetic venom variation from the different age classes of *D. siamensis* have not been completely elucidated, any alterations due to different venom compositions that could be identified as being responsible for inducing the detrimental effects of acute kidney injury after envenoming were investigated. 

### Comparative venomics and cardiovascular effects

The results indicated that the intravenously sublethal dose (0.1 mg/kg BW, IV) of juvenile, subadult or adult *D. siamensis* venom in rabbits caused changes in the systemic MAP as a biphasic response. This alteration pattern was similar to those reported in other previous *in vivo* studies using adult *D. siamensis* venom in experimental dogs [28] and rats [35], including isolated perfused kidney tissue [29]. 

The mechanism underlying the initial sudden hypotension in the first phase is most likely multifactorial and potentially involves several different processes, as reported for other snake venoms [36, 37, 38]. This change was not postulated to involve the parasympathetic (cholinergic) pathways, since an examination of sections of both vagi and atropinization in the experimental animals found no alteration of the venom action [39]. The alterations described herein can be due to a direct action of the venom on the vascular endothelium in the regulation of blood pressure or indirect release of mediators, either endogenous vasodilators or vasoconstrictors, which are fairly well-known. High proportions of PLA_2_ and SVMP by MS analysis were apparent in all three venom groups, particularly a higher amount of PLA_2_ in juvenile venom. Therefore, the sudden and significant decrease in the MAP after intravenous injection of *D. siamensis* venoms from all three venom groups was most likely mediated through the action of the two most abundant PLA_2_ and SVMP proteins in the venom (Figure 4 and Table 1). A previous study by Mitrmoonpitak et al. [40] in experimental dogs affirmed that hemodynamic changes with hypotension were induced by the effect of either PLA_2_ or SVMP components of *D. siamensis* venom. A study in anesthetized rats by Chaisakul et al. [41] described that the action of venom PLA_2_ isolated from the Papuan taipan (*O. scutellatus*) venom might play an important role in changes in the cell membrane permeability of vascular smooth muscle cells (VSM) in the development of vascular relaxation and hypotension.

The acute hypotensive response induced by *D. siamensis* venom in all venom groups in the current study does not seem to be related to the coagulopathies which are often associated with venomous snakebites, although several studies have reported that the toxic phospholipase activity of Russell’s viper venom contributes to various complications, such as the microangiopathic hemolysis, platelet-aggregation inhibitory associated with systemic neuro- and myotoxicity, disseminated intravascular coagulation (DIC) and hypotensive effects in the envenomed victims [42, 43, 44, 45, 46, 47]. These changes in coagulopathies were not apparent in the present study which may be due to the use of a smaller venom dose (sublethal dose 0.1 mg/kg BW) in each venom group, which might not provide an adequate amount of procoagulant toxins in plasma for the process of intravascular coagulation. This finding may support another study reporting that pro-coagulant activity is unlikely to be directly related to the cardiovascular collapse induced by the Eastern brown snake (*Pseudonaja textilis*) venom [48]. The question then arises of whether using a larger dose of *D. siamensis* venom can induce coagulopathies in the rabbit model, which requires further investigation. PLA_2_ toxins in *D. siamensis* venom is believed to play a role as a specific antihypertensive component through its effects on releases of thromboxane A_2_ (TXA_2_) [49], prostacyclin (PGI) [44], autacoids such as histamine [38], kinin [50], and interaction with platelets and leukocytes. However, it has been demonstrated that histamine does not appear to play a role in *D. russelii* venom-induced vasorelaxation in comparison to the presence of the histamine H1 receptor antagonist [38]. Huang [51] believed that hypotension was attributable to the properties of PLA_2_ from *Vipera russelli* venom which could release histamine from perfused guinea-pig lungs resulting in peripheral vasodilation combined with pulmonary vasoconstriction, and thus restriction of blood returning to the heart leading to a decreased cardiac output and immediately to greater hypotensive effects. The venom PLA_2_ effect involving a combination of the release of nitric oxide (NO), a vasoactive mediator for vasodilation, and thereby MAP diminution has been noted [40]. However, the mechanism behind immediate hypotension following envenoming by *D. siamensis* may be due to either the effect of PLA_2,_ or to SVMP components synergizing with a variety of other venom components that are responsible for this outcome. In addition, SVSPs components in *D. siamensis* venom may act directly on the vasculature to increase systemic vasorelaxation and immediate hypotension via the release of vasoactive mediators. It has been demonstrated that a serine proteinase isolated from the venoms of different snake species can induce vasorelaxation via the release of vasoactive mediators, for example, releasing kinin activities by *Bitis arietans* venom [50] and the vascular endothelium-derived relaxing factor by *Bothrops atrox* venom [52]. However, further studies are required to elucidate the function of SVSP from *D. siamensis* venom more precisely. A direct cardiac effect was also likely to be responsible for acute hypotension in the first phase after envenomation, despite the marked fall in blood pressure, indicating that adrenergic baroreflex responses might have masked the action of any components in the venom that induced a decreased heart rate.

The rise in the systemic blood pressure in the second phase after envenoming suggests compensatory mechanisms occurred via baroreflex responses, which was supported by the findings that the release of vasoconstrictor mediators, either catecholamines [39] or the renin-angiotensin system (RAS) [35], involves a compensatory mechanism for the hypertensive effect after envenomation with *D. siamensis*. However, there are different manners of MAP responses in the second phase among the three venom groups. The stepwise rise in MAP of either subadult or adult venom occurred to a lesser extent than in the juvenile venom and sustained hypotensions were still apparent throughout the 120-minute experimental period.

PLA_2_ toxins are ubiquitous components of snake venoms and display an array of activities. The distinctive PLA_2_ components in the juvenile venom, both acidic and basic PLA_2_, were nearly three-fold higher than those of the subadult and adult venoms which were acidic PLA_2_ according to the emPAI values (Figure 4, Table 1). Therefore, the different proportions of the two dominant PLA_2_ components may play a different significant role in the distinctive MAP increase in the second phase induced by juvenile venom, since the specific actions of the basic PLA_2_ toxins isolated from the venom of the snake *Bothrops asper* have been reported as being more effective at penetrating the phospholipid bilayer to induce permeability than acidic PLA_2_ toxins [53]. SVMP component does not rule out the marked increase in MAP induced by the juvenile venom in its action on the degradation of extracellular matrix proteins (ECM) [54,55]. Therefore, the higher abundance of both basic PLA_2_ and SVMP levels in juvenile venom might play synergistic roles in the loss of the basement membrane structure and integrity of VSMs leading to open sodium channels (ENaC) in the cells [56,57,58]. Enhanced Na^+^ influx would then cause membrane depolarization [59], which may account for the opening of calcium channels, resulting in a Ca^2+^ influx through the L-type calcium channels causing vascular contraction via the Ca^2+^-calmodulin system [60]. However, the precise contribution of juvenile venom-induced cardiovascular alterations remains to be determined. In contrast to the effects of the juvenile venom, lesser but sustained hypotensive effects from subadult and adult *D. siamensis* venoms throughout the experimental period might not rule out the effect of endogenous mediators in compensatory mechanisms. The exacerbated activation of adrenergic baroreflex mechanisms in the second phase of MAP may have been overcome by the action of various hypotensive components (enzymatic or non-enzymatic components) in the venom, leading to hypotension and bradycardia. In addition, our findings from both enzymatic activity and 2D-GE gel analysis for PDE of both adult and subadult venoms were remarkably higher compared to that of the juvenile venom. The action of PDE has been shown to play a role in hydrolysing extracellular 2’,3’-cAMP within VSMs leading to elevated adenosine and inosine levels, regulators of vascular tone [61], thus inducing vasorelaxation via mechanisms involving stimulating nitric oxide synthesis in vascular smooth muscle [62]. Taken together from these notions, the present results may suggest that a high amount of PDE in adult or subadult venoms might account for an induced vasorelaxation leading to sustained hypotension.

In the present study, the number of non-enzymatic components for KSPI, basic protease inhibitor, trypsin inhibitors (Figure 4), and vascular SVEGF (Table 1) in both subadult and adult *D. siamensis* venoms are nearly two-fold higher than in juvenile venom. These results imply that the mechanism by which the high level of these non-enzymatic components in the venoms contributes to the hypotensive effects does not involve the specific action of the SVSPs, whose amounts did not differ among the three snake venom groups. Comparative studies for the relative protein abundances of KSPIs have been reported in *D. siamensis* from Thailand (22.4%), Taiwan (28.2%) and Guangxi in China (23.2%) [63]. KSPIs are classified as basic inhibitors of proteases that have exhibited a wide variety of biological functions, including inhibition of various animal proteolytic enzymes, such as trypsin, chymotrypsin and kallikrein [64]. Protease inhibitors work by reversibly binding or interacting with proteinases, and thus influence catalytic activity [65]. 

Kallikreins in tissue and plasma are serine proteinases encoded by distinct genes, and thus differ in molecular weight, amino-acid sequence and immunogenicity. Tissue kallikrein cleaves low-molecular-weight (LMW) kininogen to produce Lys-bradykinin (Lys-BK), which is subsequently converted to bradykinin (BK) by aminopeptidase [66]. Plasma kallikrein processes high-molecular-weight (HMW) kininogen substrate to form BK. Both kinin peptides bind to the kinin B2 receptor to elicit a diverse array of biological effects, including smooth-muscle relaxation and hypotension [66]. However, there are some other possible explanations for a dual function of KSPI effects on vasorelaxation or the opposite effect on vasoconstriction *via* mechanisms involving competitive inhibition of trypsin activity on the VSM tone and selective blocking of both K^+^ and Ca^2+^ ion channels [67, 68]. The toxin members of the Kunitz superfamily have been shown to bind to voltage-sensitive ion channels, primarily K^+^ channels, and voltage-sensitive Ca^2+^ channels [68]. It is noteworthy that *D. russelii* venom has been demonstrated to induce hypotension in rodents *via* an activation of Kv and KCa channels, leading to vasorelaxation predominantly through an endothelium-independent mechanism [38]. In this context, we speculate that the high amount of KSPI in adult and subadult venoms may account for lowering blood pressure while blocking K^+^ and Ca^2+^ channels in VSMs much more potent than juvenile venom. However, to draw a firm conclusion, further studies are required to investigate the role of KSPI isolated from *D. siamensis* venom in electrophysiological experiments involving hyperpolarization of the cell membrane and relaxation of VSM tone. 

In addition, our observation of a high amount of non-enzymatic SVEGF components in the adult and subadult venoms may lead to superior hypotensive activity. Many studies have described the role of SVEGF in regulating the formation and permeability of blood vessels via mechanisms involving their interaction with kinase-linked receptors [69], enhancing vascular and capillary leakage [70], and thus contributing to hypotensive action. The higher amount of SVEGFs may induce endothelium-dependent vasorelaxation through the release of NO and PGI_2_ leading to reduced blood pressure [71]. The SVEGFs are mediators of pathological angiogenesis [72]. These effects have been recorded in patients following *D. siamensis* [73] and viperine [74] snakebites. 

It is known that the hematological effect is the first manifestation after viper snakebite and is frequently reported for hematotoxicity [75] and vasculotoxicity [76]. The SVSP family in the snake venom is responsible for the disorder and the different steps in blood coagulation and hemorrhagic behavior after a snakebite [77]. However, many of the SVSPs act as both fibrinogenolytic and fibrinolytic [77]. In the present study, the amount of SVSPs did not differ among the three snake venom groups. A direct action of SVSPs from the juvenile venom on the process of platelet agglutination would be unlikely since an abundance of α-fibrinogenases was found only in the juvenile venom. This may have an inhibitory effect on the aggregation of platelets via the degradation of the α-chain of fibrinogen [78] and fibrinogen is required for platelet aggregation by binding to the fibrinogen receptor [79]. In the present study, the RBC and PCV levels remained unchanged throughout the experiment in all venom groups in the rabbit model, a finding in contrast to the envenoming in the dog model [39, 80]. The rise in packed cell volume after envenomation in the dog model has been interpreted as baroreflex reflecting splenic contraction [80]. However, the marked absence of PCV response after treatment with *D. siamensis* venom in a rabbit model does not rule out the baroreflex effect. The differences in animal species particularly the smaller size or low storage of RBC in the rabbit spleen may cause the difference in PCV level after envenomation. 

A decrease in platelet count was apparent in all venom-treated animal groups. The initiation phase of the coagulation process occurring during Russell’s viper envenomation is partly due to the activity of the PLA_2_ component, a component of daboiatoxin [81]. PLA_2_ may hydrolyze the phospholipids of the platelet membrane and induce platelet aggregation [82]. Thus, the high amount of PLA_2_ in the juvenile venom may induce more platelet aggregation and cause a significant reduction in the platelet count throughout the experimental period. Besides the action of PLA_2_, the high amount of SVMP with a disintegrin-like domain component in the juvenile but not adult venom would inhibit platelet aggregation, while the C-type lectin family was found in the same range among all *D. siamensis* venom groups, which might not have a potent inhibitory effect on platelet aggregation in promoting different thrombocytopenia levels [83]. Furthermore, the SVMPs present mostly fibrinolytic activity. Digestion of the extracellular matrix proteins and damage to the integrity of blood vessels by SVMP cause local bleeding [84]. However, the mechanisms behind this effect have not been fully investigated and the actual relevance of the mechanism of platelet alterations induced by these venom components *in vivo* should be further clarified.

Both juvenile- and adult-venom-induced acute-phase inflammation reactions are characterized by increases in levels of lymphocytes and granulocytes at 60 minutes after administration. Decreases in monocytes were more striking in rabbits receiving juvenile and subadult venom. Intravascular hemolysis has been observed in Russell’s viper bite victims and may be due to the presence of abundant PLA_2_ in the venom that causes direct hemolysis, and the proteases then provoke hemostatic disturbances [68, 85]. Indeed, the significant increases in the MCV values and superior direct hemolysis could be attributed to the high abundance of acidic and basic PLA_2_ in the juvenile venom compared to those in the subadult and adult venoms. 

Interference in the hemostatic system leading to consumption coagulopathy has been reported to be a major clinical symptom in Russell’s viper-envenomated patients [74, 86]. In the present study, thrombocytopenia appeared in all three venom groups, among which the juvenile venom showed a greater extent of platelet reduction. The proteomic analysis of *D. siamensis* venoms indicated relatively similar amounts of both coagulation factors, X and V, activating enzymes in the venom from different aged *D. siamensis* specimens. Venom factor V and X activators may assemble on the membrane of platelets into the prothrombinase complex and then catalyze the formation of α-thrombin, initiating several positive feedback reactions that sustain its formation, with consumption of factor X, factor V, fibrinogen, and platelets. Thus, the coagulant effects of *D. siamensis* venom would be due to a thromboplastin-like action through the activation of factors X and V, which may subsequently provoke disseminated intravascular coagulation (DIC) [ 87]. 

### Venomics and renal hemodynamics effects

Intravenous injection of *D. siamensis* venoms of all venom groups showed an initial marked decrease in RBF, which coincided with a prompt fall in the systemic arterial blood pressure. Similar responses in the changes in RBF in a biphasic pattern were attributed to both the direct action of the venom on initial reduction in MAP and the subsequent coincidence with the releases of vasoconstrictor mediators causing renal vasoconstriction and thereby increasing the RVR. These observations are also in accord with previous studies of experimental dogs [39]. 

In the present study, the compensatory renal hemodynamic factors from 10 to 20 minutes after envenomation with all venom groups decreased RVR and thereby the RBF rose stepwise but remained below the control level. The juvenile venom produced different responses in renal hemodynamics, where the RBF returned to near pretreatment levels, although the systemic arterial pressure remained above the control level. However, the persistent RBF decrease induced by all venom groups suggest that changes in RBF could be mediated by either indirect activation of various vasoconstrictors [39] or the direct toxicity of *D. siamensis* venom to the kidney tissue. The extra-renal factors most likely contributing to renal vasoconstriction involve several hormonal interactions among the catecholamines, the prostaglandin systems, and RAS in modulating the changes in renal hemodynamics after intravenous administration with *D. siamensis* venom [39]. The effect of venom on DIC formation is a serious extra-renal factor that has been reported to disrupt normal blood flow to organs, especially the kidneys, and can lead to ARF in patients bitten by any of several species of snakes [88]. The phenomenon of DIC formation was not observed in the present study, whether DIC formation by *D. siamensis* venoms was produced by the activation of factors V and X in the venom causing intravascular clotting and consequently tissue ischemia and subsequently limiting the RBF. The mechanism by which *D. siamensis* venoms do not induce the formation of DIC remains elusive. The present study did not directly measure disseminated intravascular coagulation (DIC) formation after envenomation. The proteomic analysis of *D. siamensis* venoms indicated relatively similar amounts of both coagulation factors X and V activating enzymes in the venom from *D. siamensis* of different ages. The sublethal dose of venom used in the present study may be insufficient to initiate several positive feedback reactions for a thromboplastin-like action through the activation of factors X and V for subsequent provocation of DIC. This notion warrants further investigations of whether DIC formation occurs in experimental rabbits using different doses of coagulation factors (e.g factor X or V activating enzymes) extracted from *D. siamensis* venom. However, it is noteworthy that hemoglobinuria was observed in centrifuged urine samples within 10 minutes after venom injection in all groups.

The direct toxicity of *D. siamensis* venom to the kidney tissue is an important mechanism affecting changes in renal hemodynamics. Snake venoms have varying enzyme components among which the dominant protein families are PLA_2_ and SVMP and the minor protein families are LAAO and PDE [89]. The PLA_2_ enzymatic activity was significantly higher in juvenile venom, while enzyme activities of SVMP, LAAO and PDE of juvenile venom were markedly lower in comparison to subadult and adult venoms. (Figure 1B). The enzymatic activities of PLA_2_ and LAAO components between juvenile and adult *D. siamensis* venom in the present results showed similar biochemical properties of venom from *Daboia russelli siamensis* of varying ages as reported in Myanmar [4]. In previous *in vivo* studies on experimental dogs, injection of PLA_2_ or SVMP components isolated from adult *D. siamensis* venom has been demonstrated to be most injurious to the kidney causing hemodynamic changes [40]. In the present study, alterations in renal hemodynamics seemed to correlate with direct effects of the dominant protein components’ activity in the venom from each age group. The pattern of renal hemodynamics provoked by juvenile venom showed a sharper RVR decrease, starting from 30 minutes to 120 minutes of the experimental period, indicating the predominant effect of high PLA_2_ component over the several enzyme components in juvenile venom. A possible explanation for these results is based on the specific effect of PLA_2_ via overt hydrolysis of phospholipids on the cell membranes inducing lysis of endothelial cells, decreasing phosphorylation of myosin light chain resulting in vascular relaxation. In this context, vascular relaxation would decrease RVR. However, the mechanism related to intra-renal changes after envenomation would be the release of other inflammatory mediators by the kidney cells, especially platelet-activating factor (PAF). We hypothesize that *D. siamensis* envenomation *in vivo* would promote the release of PAF synthesized by kidney cells, e.g. glomerular cells, endothelial cells, renal mesangial cells and renal medullary interstitial cells, whereas release of PAF product by resting kidney cells would not be detected under basal conditions [90]. Our previous study on isolated rabbit kidney following envenomation with different components of *D. siamensis* venom [20] strengthened the findings that PAF is involved as an endogenous mediator on vasoactive parameters in the kidney. Administration of venom components isolated from adult *D. siamensis* venom for PLA_2_ and SVMP components caused an increase in the renal hemodynamics (i.e. vascular resistance and perfusion pressure). The effects of SVMP lead to increase in endogenous PAF causing vasoconstriction. However, PP and RVR were slightly increased by LAAO administration and decreased by PDE components, and thus were not directly mediated by liberation of PAF [20]. These results suggest that the alterations in renal functions induced by *D. siamensis* venom are due to the synergistic action of the different components contained in snake venom, instead of the action of a single component. In the present study, the high enzyme activity of LAAO and PDE in adult and subadult *D. siamensis* venoms compared to juvenile venom (Figure 1B) may affect the renal hemodynamics independent of the action of endogenous PAF, although PAF locally released into the kidney has been shown to play a major role as a mediator in the effect of *D. siamensis* venom on renal vascular contraction [20]. The direct effect of *D. siamensis* venom may induce inflammation and barrier damage in kidney endothelial cells by causing a localized loss of cellular homeostasis of endothelial cells, including membrane permeability to ion channel transport. Disruption in the integrity of the plasma membrane may lead to open sodium channels (ENaC) in the VSMCs [56, 57, 58]. The Na^+^ influx then causes membrane depolarization and opening of calcium channels, resulting in a Ca^2+^ influx that can then cause renal vascular contraction. The possibility could not be excluded that the appearance of constricted renal arterioles after the RBF decrease may persist until the end of the experiment. Further investigations are required to conduct experiments in isolated perfused kidney models using venoms from snakes of different ages.

The underlying mechanism of the alterations in renal hemodynamics in the liberation of PAF from kidney cells by *D. siamensis* venom may exert a receptor-mediated effect on the afferent arterioles unrelated to other vasoconstrictor mediators, either the RAS [91] or the sympathetic nervous system [92], which can interact with the formation of PAF. The interaction of PAF with these systems would not be likely, since the kidney is a major site of arachidonic acid release and its subsequent enzymatic conversion to multiple bioactive prostanoids via the cyclooxygenase metabolic pathway. Prostaglandin (PGE2) is a member of this substance group that plays an important role in renal hemodynamics. Besides exerting its effects on a specific PAF receptor within renal glomeruli, PAF dose-dependently stimulates the release of PGE2 in isolated and perfused rabbit kidneys [93, 94]. It has been reported that the different PGE2 actions are mediated via specific G protein-coupled cell surface receptors [95]. Multiple effects of PGE2 on vascular smooth muscle tonus, RBF and renal electrolytes transport have been described in rabbits [96] and microperfusion of isolated rabbit kidneys [97, 98]. Direct injection of *D. siamensis* venom into the renal artery in dogs increased the RBF and GFR mediated via the reversal of prostaglandin action by indomethacin [49]. Therefore, future studies may clarify possible participation of PGE2 in these effects induced by *D. siamensis* venom from different aged snakes in isolated perfused kidney tissue. In the present study, the effect of a low RBF causing renal ischemia after envenomation is unlikely to clarify the development of the AKI. A further reduction in renal functions to produce AKI might be expected to appear if animals were observed for a longer period of time, since the onset of ARF would range from a few to several hours after the snakebite [2]. 

### Venomics and glomerular and renal tubular effects

Envenomation with either adult or subadult venoms produced a low RBF which directly affects a decrease in both GFR and UF. The fall in GFR was accompanied by a decreased RBF of similar magnitude resulting in no alteration in the FF treated with adult or subadult venom. In contrast, juvenile *D. siamensis* venom produced comparable diminutions in GFR and UF with no systemic influences on the blood pressure, while the FF showed no significant alterations after envenomation. Local renal vasoconstriction after envenomation by juvenile, subadult or adult *D. siamensis* probably occurred in both pre-and post-glomerular arterioles, which were being influenced equally. The *D. siamensis* venom may induce the release of other mediators in renal tissue, especially the production of thromboxane B2 (TXB2), a stable metabolite of TXA2, which may synergistically enhance mesangial cell contraction and thereby sustain the reduced glomerular filtration surface and ultrafiltration coefficient (Kf). Renal changes after snakebite were expressed by mesangiolysis, glomerulonephritis, vasculitis, tubular necrosis, interstitial nephritis and cortical necrosis [99]. The marked differences in the renal histopathological changes could reflect shorter duration ischemia, a smaller dose of venom and different host response. A further reduction in the renal function might be expected to appear if the experimental animals were observed for a longer period of time.

A previous study on the histopathological changes in isolated rabbit kidneys treated with *D. siamensis* venom (10 μg/mL of perfusate) showed no preservation of renal integrity or dilation of glomerular capillary slits [29]. Destruction of the matrix structure in the glomerulus is a direct effect of *D. siamensis* venom and leads to the loss of Kf coinciding with the release of other vasoconstrictor agents and thus a decreased GFR and UF, which might be, in contrast to the *in vivo* study of all three venom groups, another mechanism for a significant and sustained reduction in both the GFR and UF. The differences in lesion presence in the glomerulus after envenomation between the *in vitro* study in the isolated rabbit kidney [20] and this *in vivo* experimental study may reflect the different venom concentrations in contact with the kidney tissue in these two models (higher venom dosage used *in vitro* than *in vivo*). The action of specific components in the venom, especially PLA_2_ and SVMP, may directly destabilize and increase the permeability of the renal glomerular cells leading to glomerular congestion, resulting in decreased GFR after envenomation. Thus, the amount of filtered load would be decreased by the filtrate delivered to the tubular segment. However, the current *in vivo* study of *D. siamensis*-envenomated rabbits showed histologically less extensive alterations in the glomerulus, which may be due to some components in the venom, such as PLA_2_, being neutralized by circulating endogenous anti-PLA_2_ proteins causing downregulation of the activity [100].

The elevations in the plasma creatinine and SDMA concentrations after envenomation in all venom groups indicated the changes in kidney function with some degree of kidney cell destruction, especially the glomerular part. The level of plasma SDMA is more reliable than creatinine as an indicator of kidney function because it is not influenced by confounding conditions. Measurement of kidney function using SDMA as a sensitive indicator [101, 102] in the present study indicated that the kidney function loss was as low as 25% after envenomation.

The present study demonstrated the effect of *D. siamensis* venom on renal tubular functions. The alterations in extrarenal factors related to renal hemodynamics (perfusion pressure and RBF), may be attributed to the limitation in renal excretion of Na^+^, K^+^, and Cl^-^ after envenomation. Herein, *D. siamensis* envenomation decreased the %FENa^+^, %FECl^-^, and osmolar clearance in all three venom age groups, with a significant effect at 30 to 120 minutes. The % FEK^+^ started to increase at 10 min after envenomation and tended to rise throughout the experimental period in all venom groups. There are some possible explanations for this difference. Virtually all the filtered electrolytes delivered to the proximal nephron, and especially Na^+^, would be reabsorbed at this site. The significance of such possible changes upon electrolyte transport in the kidney relating to the decrease/loss of renal tubular epithelial cell transport after envenomation is evident. The diminished GFR accompanying the reduced RBF could be an incidental event and itself may have a direct effect on the reduction in the filtered load of electrolytes.

An enhanced sympathetic activity has been noted after *D. siamensis* envenomation [39], which may directly influence renal tubular sodium reabsorption [103] causing a reduced urinary sodium excretion and UF. Furthermore, in this study, a reduction in electrolyte excretion and UF after envenomation occurred with vasoconstriction in the kidney, suggesting that the decreased perfusion pressure along the renal vasculature may influence the time-limited tubular transport gradient of the electrolytes leading to increased tubular reabsorption of Na^+^ and Cl^-^. Moreover, a previous study in isolated rabbit kidneys treated with *D. siamensis* venom showed a lower Na^+^ reabsorption capacity that mainly occurred in the proximal tubule [29]. Renal tubular dysfunction could be due to the synergism of specific venom components, such as SVMP and PLA_2_, causing more degradation of the plasma membrane and the ECM proteins leading to a loss of the basement membrane structural integrity [55], and thus the function of the kidney cells. However, other non-enzymatic components in the *D. siamensis* venom, such as metalloproteinase with disintegrin-like domains, and other inflammatory mediators, may be involved in the effect found in the current study.

Optical microscopy demonstrated a direct acute effect of *D. siamensis* venom on renal tubular cells, with evidence of acute tubulonephrosis of both the proximal and distal convoluted tubules, and extensive alterations in the renal tubules treated with juvenile *D. siamensis* venom (Figures 9G and 9H). Thus, alterations in the renal architecture and basement membrane structural integrity by the action of venom PLA_2_ and SVMP probably caused leaky tight junctions that connect neighboring tubular cells and allowed both Na^+^ and K^+^ to pass directly between the tubular and extracellular fluids down their concentration gradients, and thus diminished the %FENa^+^ and %FECl^-^ while augmenting the %FEK^+^. This mechanism of renal electrolyte transport by the action of *D. siamensis* venom components is supported by an extensive study of isolated perfused rabbit kidneys [20].

## Conclusions

The analysis of *D. siamensis* venom samples from specimens of three different ages by 2D-GE and MS shows that the venom proteome is altered upon ontogenetic development.We identified differentially expressed enzyme and non-enzyme components in the venom that the major ontogenetic changes appeared to be a shift in PLA_2_ component from a high concentration in juvenile venom to a lower level in adult venom and the presence of a distinct set of non-enzyme proteins for KSPI, in adult venom. We discussed the action mechanisms of venom compositional profiles among specimens from juvenile, subadult and adult *D. siamensis* correlating to cardiovascular and renal pathophysiological alterations after envenomation. Changes in renal functions after envenomation appear to be multifactorial in origin, involving changes in both extrarenal and intrarenal factors. Systemic hypotension, thrombocytopenia and hemolysis are likely to be important functional profiles among venom compositions from different age groups. A direct cytotoxic effect among venom groups on reduction of renal hemodynamics and glomerulotubular dysfunction causes acute effects on the epithelial cells of both the proximal and distal renal convoluted tubules. Juvenile venom showed the highest tubulonephrosis lesion score (scores of 0.47 and 0.27 for proximal and distal renal convoluted tubules, respectively), followed by subadult and adult (all scores were 0.13) venoms. This indicates that juvenile *D. siamensis* venom has a more nephrotoxic effect. In addition, the effect of different venom yields between juvenile and adult age groups might not outweigh the pathophysiological effects caused by the disproportionate expression of different protein families.

### Abbreviations

2D-GE: two-dimensional gel electrophoresis; ACN: acetonitrile; AKI: acute kidney injury; ARF: acute renal failure; BK: bradykinin; Cin: Inulin clearance; CPAH: PAH clearance; DIC: disseminated intravascular coagulation; ECM: extracellular matrix proteins; emPAI: exponentially modified protein abundance index; ENaC: sodium channels; FECl-: fractional chloride excretion; FEK+: fractional potassium excretiom; FENa+: fractional sodium excretion; FF: filtration fraction; GFR: glomerular filtration rate; H&E: hematoxylin and eosin; Hb: hemoglobin; HR: heart rate; In: Inulin; IV: intravenous injection; Kf: ultrafiltration coefficient; KSPI: Kunitz-type serine protease inhibitor; LAAO: L-amino acid oxidase; MAP: mean arterial blood pressure; MCV: mean corpuscular volume; MP: metalloproteinase; MS: mass spectrometry; NO: nitric oxide; PAF: platelet-activating factor; PAH: para-amino hippuric acid; PAS: Periodic Acid-Schiff; PCV: packed cell volume; PDE: phosphodiesterase; PGE2: prostaglandin; PGI: prostacyclin; PLA_2_: phospholipase A_2_; RAS: renin-angiotensin system; RBC: red blood cells; RBF: renal blood flow; RPF: renal plasma flow; RVR: renal vascular resistance; SDMA: symmetric dimethylarginine; SDS-PAGE: sodium dodecylsulphate polyacrylamide gel electrophoresis; SVEGFs: snake vascular endothelial growth factors; SVMP: snake venom metalloproteinase; SVSP: snake venom serine protease; TXA2: thromboxane A2; TXB2: thromboxane B2; UF: urine flow rate; VSM: vascular smooth muscle cells. 

## Supplementary material

The following online material is available for this article:

Additional file 1. Data on total protein identification from each pooled venom of juvenile, subadult and adult *Daboia siamensis*.
